# Production, Purification, and Characterization of a Novel Cysteine-Rich Anticoagulant from the Medicinal Leech and the Functional Role of Its C-Terminal Motif

**DOI:** 10.3390/biom15121633

**Published:** 2025-11-21

**Authors:** Valentin A. Manuvera, Ksenia A. Brovina, Vladislav V. Babenko, Pavel A. Bobrovsky, Daria D. Kharlampieva, Ekaterina N. Grafskaia, Maria Y. Serebrennikova, Nikita R. Rusavskiy, Nadezhda F. Polina, Vassili N. Lazarev

**Affiliations:** 1 Lopukhin Federal Research and Clinical Center of Physical-Chemical Medicine of Federal Medical Biological Agency, Malaya Pirogovskaya, 1a, 119435 Moscow, Russia; brovina.ka@phystech.edu (K.A.B.); daniorerio34@gmail.com (V.V.B.); pbobrovskiy@gmail.com (P.A.B.); grafskayacath@gmail.com (E.N.G.); lazar0@mail.ru (V.N.L.); 2 Center for Genetic Reprogramming and Gene Therapy, Lopukhin Federal Research and Clinical Center of Physical-Chemical Medicine of Federal Medical Biological Agency, Malaya Pirogovskaya, 1a, 119435 Moscow, Russia; 3 Moscow Center for Advanced Studies, 20 Kulakova Str., 123592 Moscow, Russia

**Keywords:** recombinant protein, anticoagulant, *Hirudo medicinalis*, antistasin, leech, *Escherichia coli*

## Abstract

The saliva of the medicinal leech *Hirudo medicinalis* contains a wide range of biologically active compounds, including multiple anticoagulants. Previously, we identified a novel cysteine-rich anticoagulant protein (CRA) from leech saliva and produced it recombinantly in *Escherichia coli*, demonstrating its potential as a basis for new anticoagulant drugs. In this study, we developed an optimized procedure for scalable production and purification of recombinant CRA. The purified protein was analyzed for common contaminants originating from *E. coli*, such as endotoxins, bacterial proteins, and DNA, and its anticoagulant properties were evaluated using standard clotting assays. Across three independent experiments, the yield of purified CRA ranged from 3.7 to 5.5 mg per liter of bacterial culture, with impurity levels per milligram of protein ranging from 7.1–31.2 ng of bacterial proteins, 1.2–15.1 ng of DNA, and 60–1445 EU of endotoxins. The purified CRA displayed electrophoretic and chromatographic homogeneity and retained strong anticoagulant activity. Additionally, a truncated form of CRA lacking the C-terminal region was produced and characterized. This variant lost membrane affinity and showed altered activity profiles, with higher thrombin time activity but reduced prothrombin time and activated partial thromboplastin time activities compared with the full-length protein.

## 1. Introduction

Cardiovascular diseases (CVDs) are a leading cause of death worldwide, with an estimated ~19.8 million deaths attributable to CVDs in 2022; the majority of these deaths are due to ischemic heart disease and stroke [1,2]. A substantial proportion of CVDs result from thrombotic processes (for example, occlusive arterial thrombosis leading to myocardial infarction or ischemic stroke), and microvascular thrombosis has also been implicated in severe forms of disease such as COVID-19–associated coagulopathy [3,4,5]. Currently, clinical practice employs several groups of effective anticoagulants, including indirect thrombin inhibitors such as warfarin, direct oral anticoagulants (DOACs) such as apixaban and edoxaban, as well as heparins and their low-molecular-weight derivatives [6]. Although these drugs are widely used and have proven efficacy, each class has notable limitations. Warfarin requires strict dietary control and frequent monitoring of coagulation parameters [7,8]; heparins can cause heparin-induced thrombocytopenia and bleeding complications [9]; and DOACs, while more convenient, may still lead to bleeding risks and are costly or contraindicated in patients with impaired renal or hepatic function [10,11]. These limitations highlight the need for new anticoagulant agents with improved safety profiles, novel mechanisms of action, or better pharmacokinetic properties. One potential source of such molecules is hematophagous animals, whose saliva typically contains a set of anticoagulants required for blood feeding. The therapeutic use of the medicinal leech (*Hirudo medicinalis*), a prominent blood-feeding species, dates back several thousand years. The saliva of the medicinal leech contains several anticoagulants, the most well-known of which is hirudin, a polypeptide competitive thrombin inhibitor [12,13]. In addition, leech saliva contains other polypeptide anticoagulants. Previously, we performed an omics analysis of the salivary gland cells of the medicinal leech and identified several novel potential anticoagulants [14]. Based on these data, we obtained a set of recombinant proteins and demonstrated that one of them—a cysteine-rich anticoagulant protein (CRA)—exhibited strong hemostasis inhibition in in vitro assays [15].

The mature form of CRA, after signal peptide cleavage, consists of 139 amino acid residues, 22 of which are cysteines. The closest known homolog of CRA is antistasin from the Mexican leech (*Haementeria officinalis*) [16]. Antistasin (ATS) and CRA share 39 identical amino acid residues. Both proteins display a characteristic pattern of cysteine residue distribution [15]. In the case of ATS, all 20 cysteine residues are involved in disulfide bond formation [17]. It is reasonable to assume that in CRA, all or nearly all cysteine residues also participate in disulfide bond formation.

We previously established that CRA exhibits a pronounced ability to inhibit coagulation in in vitro clotting assays [15], which are commonly used in clinical practice to evaluate the hemostatic system based on fibrin clot formation time [18]. To more precisely assess this effect, we modified the standard methodology of three clinical clotting assays for testing anticoagulant solutions rather than patient serum parameters. We employed activated partial thromboplastin time (aPTT), prothrombin time (PT), and thrombin time (TT) assays [19]. In all three assays, CRA demonstrated a clear dose-dependent inhibition of coagulation. Notably, in the TT assay, its effect was weaker than that of hirudin (Hir), whereas in PT and especially aPTT assays, CRA was significantly more potent than Hir. This suggests a difference in the mechanism of hemostasis inhibition between the two proteins.

Previously, we obtained CRA using a preliminary simplified protocol [15]. It included bacterial cell lysis, washing of the insoluble fraction, solubilization in buffer containing 8 M urea, metal chelate chromatography under denaturing conditions, and dialysis. This approach allowed isolation of small amounts of protein sufficient for preliminary analysis. The purity of the preparation was not controlled, and electrophoretic analysis revealed numerous impurities. Gel filtration analysis indicated a high content of high-molecular-weight aggregates. Protein losses during dialysis reached 80–90%, and the total yield did not exceed 1–2 mg per liter of bacterial culture. Moreover, purification from such contaminants as bacterial endotoxins, *E. coli* DNA, and host proteins was insufficient, which is critical for testing CRA in in vivo experiments and for its potential use in medical formulations. In this study, we describe a high-yield method for obtaining the cysteine-rich anticoagulant from the medicinal leech with a high degree of purity.

## 2. Materials and Methods

### 2.1. The Construction of the Expression Plasmid

The plasmid carrying the recombinant gene encoding CRA was constructed using the PIPE method [20]. The plasmid pET-22b(+) (Novagen, Darmstadt, Germany) was amplified by PCR in its entirety, except for the region encoding the pelB signal peptide. The oligonucleotides PETPIPE-1N (5′-CATATGTATATCTCCTTCTTAAAGTTAAAC-3′) and PETPIPE-2N (5′-CTCGAGCACCACCACCACCACCACTGAGATC-3′) were used for this purpose. The DNA fragment encoding the mature form of CRA without the signal peptide was amplified by PCR using oligonucleotides CRApipf (5′-GGAGATATACATATGGACGAAGAAAACTATGAAGATG-3′) and CRApipr (5′-GTGGTGGTGCTCGAGATTCTTTTTCCCTTTGAAGAAATTC-3′). The cDNA from *H. medicinalis* salivary gland cells, previously obtained from laser-microdissected material [14], served as the amplification template. The resulting amplicons were purified by agarose gel electrophoresis and mixed in a 1:1 mass ratio. The resulting mixture, without ligation, was used to transform *Escherichia coli* TOP10 cells (Invitrogen, Thermo Fisher Scientific, Waltham, MA, USA), which were plated on LB agar plates containing ampicillin (150 mg/L). Colonies carrying plasmids with the target DNA fragment were identified by PCR. Selected colonies were cultured, and plasmid DNA was isolated from the biomass. The structure of the DNA insert in the plasmids was verified by Sanger sequencing on an automated sequencer. As a result, the plasmid pET-hmCRA was obtained. This plasmid carries the recombinant gene under the control of the T7 promoter, encoding the mature form of CRA (without the signal peptide) fused with a C-terminal His-Tag (Figure S1).

**Figure 1:**
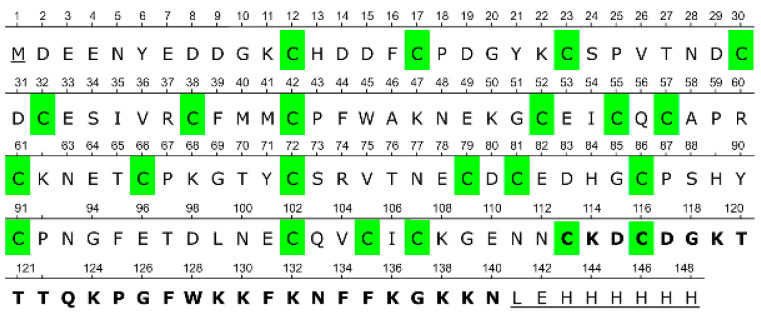
Full amino acid sequence of the recombinant CRA. Amino acid residues additionally introduced relative to the native protein are underlined; residues deleted in the truncated CRA-cut protein are shown in bold; cysteine residues are highlighted in green.

The DNA fragment encoding the truncated form of CRA lacking the C-terminal region (CRA-cut) was obtained by PCR using oligonucleotides T7 (5′-TAATACGACTCACTATAGGG-3′) and CRAcutR (5′-GTGCTCGAGATTATTTTCTCCTTTGCAGATGC-3′). The plasmid pET-hmCRA served as the amplification template. The resulting fragment was sequentially treated with the restriction endonucleases XbaI (Thermo Fisher Scientific, Waltham, MA, USA) and XhoI (Thermo Fisher Scientific, Waltham, MA, USA). The plasmid pET-22b(+) was treated in the same manner. After agarose gel purification, the obtained vector and insert fragments were mixed at a molar ratio of 1:8 in ligation buffer supplemented with T4 bacteriophage ligase (Thermo Fisher Scientific, Waltham, MA, USA) and incubated at room temperature for one hour. The resulting mixture was used to transform *E. coli* TOP10 cells, which were plated on LB agar containing ampicillin (150 mg/L). Similarly to the construction of the pET-hmCRA plasmid, clones carrying plasmids with the target DNA fragment were selected, and the inserted DNA encoding the protein of interest was sequenced. As a result, the plasmid pET-hmCRA-cut was obtained, which encodes CRA lacking 28 C-terminal amino acid residues. In all other respects, it is identical to pET-hmCRA (Figure 1).

### 2.2. Cultivation of the Producer Strain

*E. coli* BL21(DE3) gold cells (Novagen, Darmstadt, Germany) were transformed with the plasmid pET-hmCRA or pET-hmCRAcut. Transformants were plated on LB agar plates containing ampicillin (150 mg/L) and incubated for 16 h at 37 °C. A single bacterial colony was transferred into an LB medium containing ampicillin (150 mg/L) and cultivated in a shaker incubator at 37 °C for six hours. The resulting culture was used to inoculate an autoinduction TB medium (24 g/L yeast extract, 12 g/L tryptone, 4 g/L glycerol, 0.17 M KH_2_PO_4_, 0.72 M K_2_HPO_4_, 150 mg/L ampicillin, 1 g/L glucose, 0.5 g/L lactose) at a ratio of 1:100. Culture flasks (1.5 L) containing 250 mL of inoculated medium were incubated for 18 h in a shaker incubator at 220 rpm and 37 °C. Cells from the overnight culture were harvested by centrifugation for 15 min at 5000× *g*. The pellet was stored at −20 °C.

### 2.3. Isolation of CRA

The cell pellet obtained in the previous step was resuspended in US buffer (PBS, 0.1% Triton X-100, 0.1 g/L lysozyme, 5 mM EDTA) at a ratio of 100 mL of US buffer per 1 L of initial culture and disrupted using a Branson 250 Sonifier (Branson Ultrasonics, Danbury, CT, USA) at 22 kHz for 10 min on ice. The lysate was centrifuged for 15 min at 15,000× *g*. The resulting pellet was resuspended in 2% Triton X-100 using a sonifier for 1 min on ice and centrifuged again for 5 min at 10,000× *g*. The detergent washing step was repeated twice. The washed pellet was solubilized in buffer AM (8 M urea, 20 mM Tris-Cl, 500 mM NaCl, 10 mM imidazole, pH 7.5) at a volume of 1/10 of the initial bacterial culture using sonication at 22 kHz for 5 min on ice. The insoluble residue was removed by centrifugation for 30 min at 50,000× *g*. The obtained solution was loaded onto an HR16/20 column containing 10 mL of Ni Sepharose Fast Flow (Cytiva, Marlborough, MA, USA) equilibrated with buffer AM. After loading, the column was washed with 10 mL of AM buffer, then with 50 mL of wash buffer BM (8 M urea, 20 mM Tris-Cl, 500 mM NaCl, 25 mM imidazole, pH 7.5), and eluted with buffer EM (8 M urea, 20 mM Tris-Cl, 500 mM NaCl, 500 mM imidazole, pH 7.5).

Refolding of CRA was performed by rapid 20-fold dilution in 20 mM Tris-Cl, pH 7.5, at 4 °C. The cooled Tris-Cl solution was poured into a beaker and placed on a magnetic stirrer for vigorous mixing. Then, the CRA protein solution in EM buffer obtained in the previous step was quickly added. The beaker was sealed with parafilm and incubated for 16–18 h at 4 °C without stirring. The precipitate formed was removed by centrifugation for 30 min at 50,000× *g*.

For purification, the refolded protein solution was applied to a Tricorn10/50 column containing 4.5 mL of DEAE Sepharose FF (Cytiva, Marlborough, MA, USA). The column was pre-equilibrated with 20 mM Tris-Cl, pH 7.5, and washed with 10 mL of the same buffer after sample loading. Elution was carried out with a linear NaCl gradient from 20 mM Tris-Cl, pH 7.5, to 20 mM Tris-Cl, 1 M NaCl, pH 7.5, over 50 mL. Chromatography was performed using an NGC chromatograph (Bio-Rad, Hercules, CA, USA) at a flow rate of 2 mL/min. Process control and fraction collection were based on absorbance at 280 nm. The presence of the target protein in the fractions was confirmed by SDS-PAGE with Coomassie G-250 staining. Fractions containing CRA were combined and dialyzed against 100 volumes of water. The resulting protein solution was aliquoted, frozen, and lyophilized for 20 h. Dried samples were stored at −20 °C.

### 2.4. Isolation of CRA-Cut

The cell pellet was resuspended in buffer AN (20 mM Tris-Cl, 500 mM NaCl, 10 mM imidazole, pH 7.5) at a ratio of 100 mL per 1 L of culture and disrupted using a Branson 250 Sonifier (Branson Ultrasonics, Danbury, CT, USA) at 22 kHz for 10 min on ice. The lysate was centrifuged for 15 min at 15,000× *g*. The pellet was discarded, and the supernatant was heated to 65 °C and centrifuged for 10 min at 15,000× *g*. The pellet of denatured proteins was discarded, and the supernatant was centrifuged again for 30 min at 50,000× *g*. The resulting solution was applied to an HR16/20 column containing 10 mL of Ni Sepharose Fast Flow (Cytiva, Marlborough, MA, USA) equilibrated with buffer AN. After loading, the column was washed with 10 mL of AN buffer, then 50 mL of wash buffer BN (20 mM Tris-Cl, 500 mM NaCl, 25 mM imidazole, pH 7.5), and eluted with buffer EN (20 mM Tris-Cl, 500 mM NaCl, 500 mM imidazole, pH 7.5). Fractions containing the protein of interest (POI) were combined and diluted fivefold with 20 mM Tris-Cl, pH 7.5. CRA-cut was further purified using IEX chromatography as described for full-length CRA. The purified CRA-cut solution was stored at −20 °C.

### 2.5. Protein Concentration Determination

Lyophilized protein was dissolved in water for 30 min with stirring. CRA concentration was determined by measuring absorbance at 280 nm. The calculated molar and mass extinction coefficients for CRA were 19,140 M^−1^cm^−1^ and 1.11 mg^−1^cm^−1^, respectively. For CRA-cut, the calculated molar and mass extinction coefficients were 13,210 M^−1^cm^−1^ and 0.96 mg^−1^cm^−1^, respectively.

### 2.6. Clotting Assays

The APG4-03-Ph (EMCO LLC, Moscow, Russia) hematology analyzer was used for coagulation assays. All measurements were performed using control plasma (Renam, Moscow, Russia). Activated partial thromboplastin time (aPTT), prothrombin time (PT), and thrombin time (TT) were determined as described previously [19]. Each sample was measured in four replicates in parallel using four coagulometer measurement cells. CRA concentrations of 1 µM were used for aPTT and PT assays, and 1 µM and 5 µM for TT assays. Concentrations of anticoagulant proteins are indicated for the initial sample. In the reaction mixture, the concentration is six times lower in the case of aPTT (25 µL from 150 µL), four times lower in the case of TT (50 µL from 200 µL), and three times lower in the case of PT (50 µL from 150 µL).

### 2.7. Determination of Impurities in Protein Samples

The content of *E. coli* proteins in CRA samples was determined by ELISA using the Enzyme-linked Immunosorbent Assay Kit for *Escherichia coli* Host Cell Protein Residue (Cloud Clone Corp., Katy, TX, USA). *E. coli* DNA content was determined by quantitative real-time PCR using the *E. coli* Host Cell DNA Quantitation Kit (Biolabmix, Moscow, Russia). Endotoxin levels were measured using the chromogenic endpoint LAL assay (BioEndo, Chengdu, China). All measurements were performed according to the manufacturers’ instructions.

### 2.8. Analytical Gel Filtration

Analytical gel filtration was performed using an NGC chromatograph (Bio-Rad, Hercules, CA, USA) on a Tricorn 10/300 column (Cytiva, Marlborough, MA, USA) packed with Superdex 200 (Cytiva, Marlborough, MA, USA). The column was equilibrated with PBS (20 mM sodium phosphate, 8 g/L NaCl, pH 7.4). Calibration was performed using the LMW Gel Filtration Calibration Kit (Cytiva, Marlborough, MA, USA). The flow rate was 1 mL/min, and protein elution was monitored at 280 nm.

### 2.9. Protein Binding with Lipid Vesicles

Giant unilamellar vesicles consisting of Chol:PC:PE:PS (1:3:2:2) were used to mimic endothelial cell membranes [21]. The vesicles were prepared and kindly provided by Dr. P.V. Bashkirov. 150 µL of liposomes (1 mg/mL) were mixed with 50 µL of protein solution (1 mg/mL) and incubated for 15 min at room temperature. The mixture was centrifuged for 10 min at 12,000× *g*, and the supernatant was transferred to another tube. The pellet was washed with saline and dissolved in 15 µL of 1% SDS. Samples were analyzed by SDS-PAGE and stained with Coomassie G-250. From the supernatant fraction, 5 µL was loaded onto the gel, while the entire pellet fraction was loaded in full.

### 2.10. Modeling of the CRA Spatial Structure

The AlphaFold 3 [22] software was used for protein structure prediction. The AlphaFold protein model was visualized with UCSF ChimeraX 1.7 [23].

### 2.11. Circular Dichroism (CD) Spectroscopy

For CD spectroscopy-based analysis, the proteins were diluted to 0.2 mg/mL in H_2_O. The CD spectra were registered on a Chirascan VX Spectrophotometer (Applied Photophysics Ltd., Leatherhead, UK) in cuvettes of 0.05 cm optical path in two repeats and averaged after baseline subtraction. The spectra were analyzed using CDNN software version 2.1 (Applied Photophysics Ltd., Leatherhead, UK) with default settings.

## 3. Results

### 3.1. Isolation of Recombinant Proteins

During heterologous expression in *E. coli*, CRA actively accumulated in the cells and remained in the pellet fraction after lysis. We performed three completely independent CRA isolations—from bacterial transformation to lyophilization. Each isolation was carried out from 1.5 L of bacterial culture. The resulting samples were analyzed for anticoagulant activity, as well as for the content of producer strain proteins, DNA, and endotoxins. In addition, the oligomeric status of the proteins was determined using analytical gel filtration.

**Figure 2:**
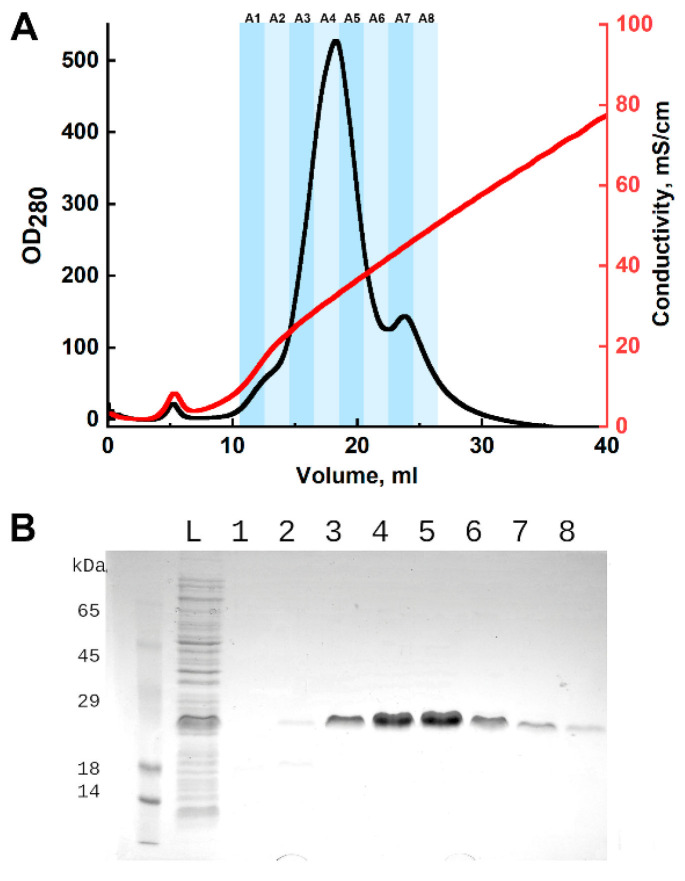
Purification of CRA by ion-exchange chromatography. (**A**)—chromatographic profile: the *x*-axis represents elution volume, left y-axis represents optical density at 280 nm, and right y-axis represents conductivity. Fractions are indicated by shaded areas and labeled above. (**B**)—SDS-PAGE analysis of fractions with Coomassie G-250 staining. L—washed cell pellet lysate; 1–8—chromatographic fractions, numbering corresponds to the chromatogram above. The predicted molecular weight of the CRA protein is 17.2 kDa.

As a result of three independent isolations, 5.62 mg, 8.2 mg, and 8.3 mg of CRA were obtained. SDS-PAGE analysis demonstrated electrophoretic homogeneity of the samples (Figure 2). Upon dissolution of the lyophilized protein, it was completely solubilized, which was confirmed by the absence of precipitate after centrifugation and by the equality of protein concentrations before lyophilization and after reconstitution.

CRA-cut, in contrast to CRA, was detected in the soluble fraction of the *E. coli* lysate. After optimizing the purification conditions, we carried out a preparative purification of CRA-cut from 1.5 L of bacterial culture (Figure S2). As a result, 12.7 mg of purified CRA-cut was obtained.

### 3.2. Determination of Activity

The results of aPTT, PT, and TT assays are shown in Table 1. For each point, four independent measurements are conducted; the table presents the mean value with the standard deviation. The p-values for multiple group comparisons are provided in the Supplementary Materials (Table S1). In all tests, the time of the measured groups significantly differs from the control groups without anticoagulants. It is also worth noting the presence, in a number of cases, of significant differences in activity between the three CRA preparations, although ideally the values should be identical. The most active protein preparation, CRA P1, showed significantly longer clotting times than the other two preparations in both the aPTT and PT assays. Only in the TT assay the difference between CRA P1 and CRA P3 was not statistically significant. In the TT assay preparation CRA P3 also showed no significant difference from CRA P2. These results may indicate the presence of real differences in specific activity between individual protein preparations.

### 3.3. Determination of Impurities

Three independent CRA preparations (P1–P3) were obtained under identical expression and purification conditions, and the levels of *E. coli* host cell proteins, DNA, and lipopolysaccharides (LPS, endotoxins) were determined (Table 2).

The total yield of CRA ranged from 5.6 mg to 8.3 mg per preparation, indicating reproducible expression and purification efficiency. The content of *E. coli* host cell proteins was low, varying between 7.1 ± 0.4 ng/mg and 31.2 ± 1.9 ng/mg, which corresponds to a high level of purification. Residual *E. coli* DNA was detected in the range of 1.2 ± 0.1–15.1 ± 2.4 ng/mg, with some variation between preparations. The endotoxin (LPS) content showed the largest differences, from 60.0 ± 2.8 EU/mg to 1445 ± 102 EU/mg, despite identical purification conditions. This variability likely reflects biological differences between production batches rather than procedural inconsistencies. Overall, the obtained CRA preparations demonstrated consistent yields and a high degree of purity, although the variation in endotoxin and DNA levels indicates the need for careful monitoring of these parameters during production.

### 3.4. Analytical Gel Filtration

**Figure 3:**
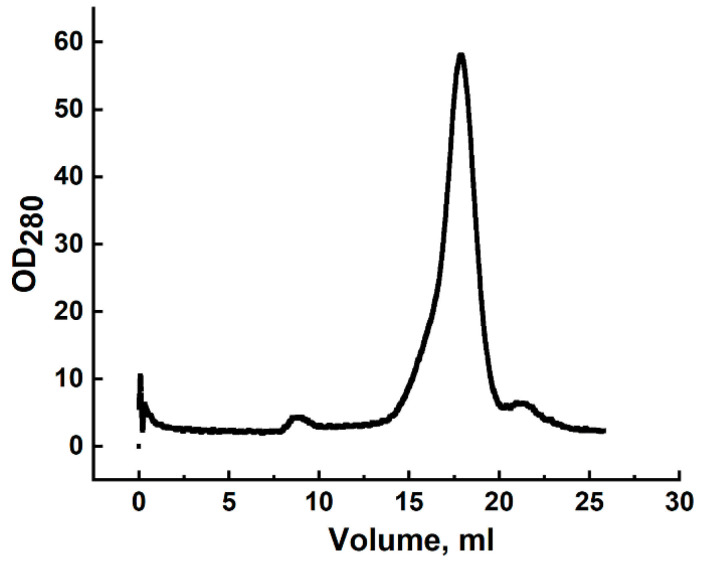
Gel filtration profile of CRA refolded by rapid dilution and purified by DEAE-Sepharose chromatography using a Superdex 200 column. The *x*-axis represents elution volume; the y-axis shows optical density at 280 nm.

Gel filtration of all three samples yielded a single peak (Figure 3), with apparent molecular masses of 24.5 kDa, 24.9 kDa, and 25.1 kDa, respectively.

### 3.5. Binding of Proteins to Lipid Vesicles

**Figure 4:**
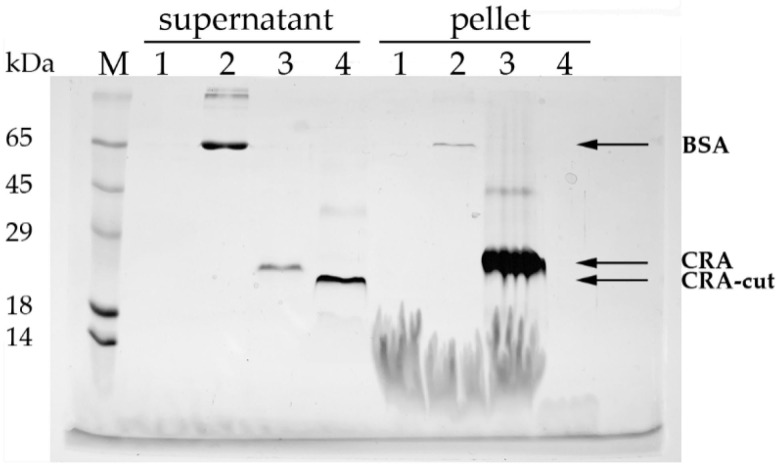
Binding of proteins to lipid vesicles. SDS-PAGE analysis (Laemmli) with Coomassie G-250 staining. M—molecular weight markers; 1—vesicles without protein; 2—BSA; 3—CRA; 4—CRA-cut; supernatant and pellet fractions are labeled on the gel. The predicted molecular weight of the CRA protein is 17.2 kDa, and that of CRA-cut is 13.8 kDa.

The binding of CRA, CRA-cut, and BSA (control) to lipid vesicles was investigated. It was found that CRA effectively binds to vesicles and sediments with them during centrifugation, whereas CRA-cut was not detected in the pellet and remained entirely in the supernatant (Figure 4).

### 3.6. Modeling of the CRA Spatial Structure

**Figure 5:**
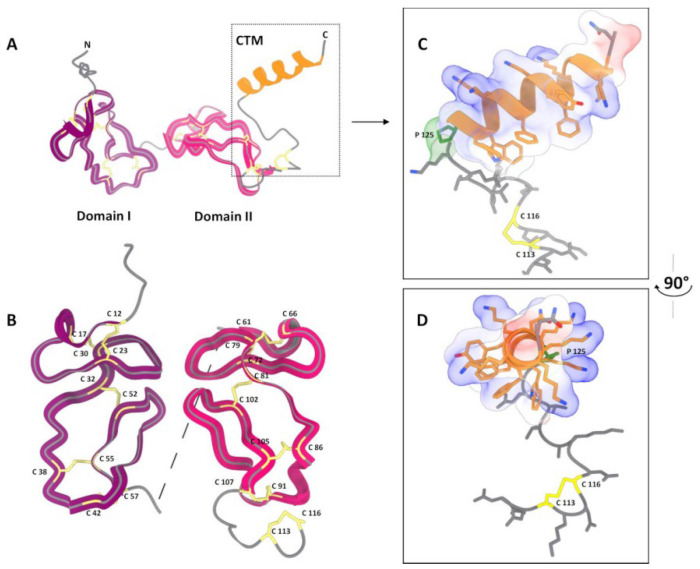
Visualization of the CRA spatial structure modeling using AlphaFold3 [22]. (**A**)—Overall polypeptide chain fold. Two well-defined domains and a separate C-terminal helical motif (CTM) are visible. (**B**)—Comparison of the structure of the two domains. (**C**,**D**)—Structure of the CTM in two projections. Protein model was visualized with UCSF ChimeraX 1.7 [23].

The overall architecture of CRA is similar to that of antistasin (Figure 5A). The pLDDT plots and PAE matrix are presented in the Supplementary Materials (Figure S5). It has two clearly defined homologous structural domains, each cross-linked by five disulfide bonds (Figure 5B). However, there are two notable differences. First, the interdomain spacer in CRA is much shorter than in antistasin and consists of only three amino acid residues with proline in the center—Ala-Pro-Arg. In antistasin, the interdomain spacer also contains a proline residue in the middle but consists of eight residues (Arg-Leu-Glu-Pro-Met-Lys-Ala-Thr). This likely results in a more rigid relative fixation of the domains in the CRA molecule. Second, CRA possesses a large helical C-terminal motif (CTM). Furthermore, due to the alternation of residues in the primary structure, the sides of the helix have different properties. Predominantly Lys residues are grouped on one side, giving it a pronounced positive charge. On the opposite side, primarily aromatic hydrophobic groups are clustered (Figure 5C,D). The base of the CTM contains a disulfide bond (Cys113-Cys116) and Pro125. Such a motif apparently ensures a specific orientation of the CTM relative to the CRA molecule.

### 3.7. Circular Dichroism (CD) Spectroscopy

CRA and CRA-cut show similar CD spectra, which are provided in the Supplementary Materials (Figure S3). Both spectra have a single negative peak at a wavelength of approximately 200 nm. This indicates a predominance of loops and coils in the protein structure, with a lack of significant amounts of regular secondary structure elements such as alpha-helices and beta-sheets [24,25]. This result is entirely consistent with the structure of anistasin, which lacks helices and sheets and consists mainly of loops stabilized by numerous disulfide bonds [17]. Bdellastasin, a single-domain homolog of antistasin, has a CD spectrum very similar to that of CRA, for both the native and recombinant protein [26].

## 4. Discussion

The cysteine-rich anticoagulant (CRA) of the medicinal leech contains a large number of cysteine residues, which is reflected in its name. Antistasin, the closest homolog of CRA for which a spatial structure has been determined [17], also contains a high number of cysteine residues, all of which participate in disulfide bond formation. ANS possesses a unique fold that lacks canonical regular elements of secondary structure, but has numerous loops stabilized by disulfide bridges at their bases, which maintain its structure. Such structural features do not inspire optimism when expressing the recombinant protein in *E. coli*. The intracellular environment of *E. coli* has a reducing redox potential, which greatly impedes disulfide bond formation [27], and cytoplasmic proteins with disulfide bonds are virtually absent in *E. coli* [28]. In general, obtaining active recombinant proteins containing multiple disulfide bonds in *E. coli* is a rather challenging task [29,30,31]. However, in the case of CRA, we were able to obtain an active protein without employing sophisticated methods or complicated approaches. The reason undoubtedly lies in the strong intrinsic ability of CRA to form its tertiary structure during in vitro folding. Previously, we only described a crude isolation of CRA using metal-chelate chromatography without additional purification [15]. We performed refolding by dialysis, which led to very high losses (80–90%) and a final yield of the target protein of no more than 1–2 mg per liter of bacterial culture. First, we optimized new refolding conditions. We used dialysis and rapid dilution at pH values from 5 to 10 at room temperature and +4 °C. As a result, we found that the best result is achieved with rapid dilution using a cold 20 mM TrisCl pH 7.5 solution. Under these conditions, no visible protein precipitate forms and almost all the protein remains in solution. Second, we introduced an additional purification step using ion-exchange chromatography (IEX). The calculated isoelectric point for CRA is 5.7. Based on this, we performed chromatography on two anion-exchange sorbents—DEAE-sepharose and Q-sepharose—with elution by a linear NaCl gradient. The results were similar, but in the case of DEAE-sepharose, elution occurred at lower NaCl concentrations, so we used this option thereafter. The IEX step allowed us to combine additional protein purification with its concentration after refolding by rapid dilution. Following this, we performed three independent purifications of CRA and measured the activity of the obtained samples as well as the levels of three types of impurities: *E. coli* proteins, *E. coli* DNA, and endotoxins. The level of contaminating proteins was low and generally within acceptable pharmacological limits (which is commonly targeted below 100 ng per mg of final product), although this parameter did not strictly indicate by FDA and depend in each case on host cell type, the immunogenicity of specific host cell proteins and the administered dose [32]. The level of lipopolysaccharides was relatively low but showed significant variation among the samples. This parameter requires specific monitoring during CRA purification and, most likely, additional purification steps, for example, using polymyxin-Sepharose [33,34]. The level of host DNA was relatively high [35], which may also require further purification or adjustment of chromatographic conditions if CRA is to be used internally as an anticoagulant. For research purposes, however, the level of purity can be considered satisfactory. Thus, the proposed method of CRA production provides a high degree of purity and allows additional purification steps to be planned depending on the intended use of the protein.

In all three CRA preparations, the clotting assays demonstrated a high level of anticoagulant activity. However, the variability among samples was quite significant (Table 1, Supplementary Materials (Table S1)). We assume this variability is related to protein refolding conditions. Although we attempted to reproduce the same conditions in all three cases as precisely as possible, a number of uncontrolled parameters remain. One of them may be the degree of oxygen saturation of the dilution buffer used for rapid dilution. Other factors that could affect the results include the duration of purification, storage time of the frozen biomass (ranging from several hours to several days), and other aspects that became apparent only after analyzing the anticoagulant activity of the samples. Therefore, in the case of large-scale production, we believe that the refolding stage requires particularly careful control.

During optimization of the cultivation and purification procedures for CRA, we noticed some peculiarities. When analyzing a fresh culture during growth by SDS-PAGE, CRA was always detected entirely in the soluble fraction of the cell lysate. However, during preparative lysis, the protein shifted to the pellet fraction. This may indicate either protein denaturation or its association with sedimented cell membranes, as we previously hypothesized [15]. Supporting the latter assumption is the fact that washing the pellet with Triton X-100 gradually released CRA into the soluble fraction. For this reason, we chose two washing steps, at which point target protein losses were still minimal. Moreover, although difficult to formalize, the washed pellet from the lysate did not resemble classical inclusion bodies, which typically form a milky-white suspension. In this case, the pellet was much more similar to a membrane fraction.

We made several attempts to isolate a soluble form of CRA to avoid the denaturation–renaturation process. CRA could not be purified from the soluble lysate fraction at all. CRA could be purified from the Triton X-100 wash fractions, but with low and unstable yield. The average activity of the obtained protein in aPTT assays did not differ from that of the refolded protein. Therefore, we ultimately used the purification scheme described above.

Unlike antistasin, CRA possesses, in addition to two cysteine-rich domains, an additional C-terminal segment consisting of 28 amino acid residues, counting from the first of two cysteines present in this region [15]. This motif is composed mainly of alternating hydrophobic residues and ten lysine residues. We hypothesized that this motif mediates CRA binding to cell membrane lipids. The results of CRA folding modeling using AlphaFold are consistent with our predictions based on the analysis of its amino acid sequence. The protein’s structure is similar to antistasin (Figure 5A). It is intriguing that its two domains exhibit mirror symmetry (Figure 5B). However, it is important to remember that this is the result of in silico modeling, not supported by experimental data, and should be treated with a certain degree of caution. The CTM forms a structure distinct from the core of the protein molecule. Its orientation is determined by the disulfide bond and the proline residue present at the base of the motif. The asymmetry of the CTM helix sides results in one surface carrying a positive charge due to Lys residues, while hydrophobic aromatic side groups are clustered on the opposite surface. We hypothesize that this particular feature of the CTM enables its binding to membrane phospholipids. The logical next step was to obtain a truncated form of CRA lacking the C-terminal motif (CRA-cut) and to examine how this modification affected its properties. The results were somewhat surprising. Regarding membrane affinity, our hypothesis was confirmed: CRA-cut accumulated and was purified from the water-soluble fraction of the *E. coli* lysate. The yield of the protein was higher than that of the full-length form, mainly due to product losses during refolding. The full-length CRA bound efficiently to lipid vesicles, whereas CRA-cut did not show such affinity (Figure 4). Unexpectedly, the results of the coagulation assays revealed that CRA-cut performed worse than CRA in aPTT and PT tests, but significantly better in the TT test (Table 1).

We assume that this intriguing effect may be due to the C-terminal motif acting as a modifier of CRA binding specificity toward inhibited proteases of the coagulation cascade, similar to how staphylokinase alters plasmin substrate specificity [36]. Certain difficulties arise in the interpretation because coagulation assays are integral tests. The aPTT test is used to measure coagulation parameters during activation of the intrinsic pathway, the PT test assesses the extrinsic pathway, and the TT test measures fibrinogen activation by thrombin. A thrombin inhibitor will show activity in all three tests, as observed for hirudin. CRA-cut increases TT while simultaneously decreasing aPTT and PT compared to CRA. This effect can only be explained by the increased affinity of the truncated form for thrombin and a concomitant strong decrease or loss of affinity for other proteases upstream in the coagulation cascade. Unfortunately, the specific components of the hemostasis cascade, apart from thrombin, that are affected by CRA remain unknown. To clarify this issue, we performed chromogenic assays for the inhibition of factors Xa, XIa, XIIa, and IIa (thrombin). The measurement conditions are described in the Supplementary Materials (Figure S4). It was completely unexpected that CRA showed no inhibition in any of the tests. One could conclude from this that CRA is not a direct inhibitor of these factors; however, in the case of thrombin, there is a strong argument against such a conclusion. We have previously shown that in a system containing purified thrombin and fibrinogen, CRA effectively inhibits clot formation [15]. We repeated this experiment, as well as the clotting test for factor Xa inhibition (Supplementary Materials (Figure S4A)). The obtained results confirmed our previous findings. In clotting tests, CRA demonstrates inhibition of both thrombin and factor Xa. In the thrombin inhibition clotting test, CRA and CRA-cut showed identical results (Supplementary Materials (Figure S4B)). In the factor Xa inhibition test, CRA showed higher activity (Supplementary Materials (Figure S4A)). However, it is important to consider that these tests are not direct. In the factor Xa inhibition test, the effector is also thrombin, which is itself inhibited by CRA. Furthermore, these tests show weak dose-dependence (Supplementary Materials (Figure S4)). Consequently, the informativeness of such measurements is extremely limited. Thus, two hypotheses are possible. The first is that the chromogenic substrates used are able to access the active site of the coagulation factors during their competitive inhibition by CRA. This could be explained by the much lower molecular mass of these substrates compared to the mass of the proteins cleaved by the factors. If the contact between the inhibited enzyme and CRA is not sufficiently tight, the low-molecular-weight substrate could diffuse into the enzyme’s active site. If this is the case, then chromogenic tests using short peptide substrates are inapplicable here. The other possibility is that the inhibition mechanism is more complex than direct binding. Perhaps blood plasma or insufficiently purified fibrinogen contains some additional components necessary for CRA’s function. We currently have no experimental evidence to favor either version.

The ability of the CTM to bind membranes is also of interest. Coagulation factors themselves exhibit affinity for vesicles [37]. The extrinsic coagulation pathway is initiated upon vessel wall injury, partly due to the damage of epithelial cell membranes [38]. All these observations theoretically allow for several functional possibilities for CRA. For instance, the CTM may act as a targeting element, increasing the local concentration of CRA at the site of vessel injury or on vesicles associated with coagulation factors. It is also possible that CRA binds to platelets. Experimental confirmation of which of these scenarios is realized in vivo is currently lacking and remains a topic for further investigation.

## 5. Conclusions

We obtained a novel recombinant cysteine-rich anticoagulant from the leech *Hirudo medicinalis* and described a method for its expression and purification. The recombinant protein exhibits high anticoagulant activity in standard assays for activated partial thromboplastin time (aPTT), prothrombin time (PT), and thrombin time (TT). We also obtained a truncated form of the protein lacking the protruding C-terminal motif. The truncated form lost its affinity for lipid membranes. At the same time, its activity in aPTT and PT assays decreased, while its TT activity was significantly higher than that of the full-length protein. We assume that the C-terminal motif acts as a modifier of the binding specificity of CRA toward the proteases it inhibits.

## Figures and Tables

**Table 1:** Determination of the activity of purified CRA using aPTT, PT, and TT tests.

Sample	aPTT, 1 µM		PT, 1 µM		TT, 1 µM		TT, 5 µM	
	Time, s	S/C	Time, s	S/C	Time, s	S/C	Time, s	S/C
CRA P1	151.5 ±13.5	5.7	87.6 ± 2.3	5.3	21.8 ± 0.4	1.5	66.8 ± 9.6	5.7
CRA P2	53.3 ± 2.5	2	30.4 ± 1.4	1.8	16.6 ± 0.1	1.2	30.7 ± 3.2	2.6
CRA P3	101.2 ± 3.8	3.8	57.5 ± 2.6	3.5	20.1 ± 0.1	1.4	43.3 ± 5.1	3.7
CRA-cut	36.0 ± 1.9	1.4	20.7 ± 0.2	1.2	22.6 ± 1.8	1.6	103.9 ± 7.3	8.9
C	26.4 ± 0.4	1	16.5 ± 0.5	1	14.3 ± 0.4	1	11.7 ± 0.1	1

CRA P1, P2, and P3—three independent CRA isolations; C—control sample without protein. Data are presented as mean ± standard deviation; measurements for each group were performed in four replicates (*n* = 4). S/C—ratio of sample clotting time to control.

**Table 2:** Content of *E. coli* proteins, DNA, and lipopolysaccharides (endotoxins) in purified CRA samples.

Sample	CRA Yield, mg	*E. coli* Proteins, ng/mg	*E. coli* DNA, ng/mg	LPS, EU/mg
CRA P1	5.6	7.1 ± 0.4	8.1 ± 2.1	1445.0 ± 102.1
CRA P2	8.2	31.2 ± 1.9	15.1 ± 2.4	60.0 ± 2.8
CRA P3	8.3	30.6 ± 1.9	1.2 ± 0.1	146.2 ± 5.8

CRA P1, P2, and P3—three independent CRA isolations; CRA amount—total yield in mg; *E. coli* proteins—impurity protein content in ng per 1 mg CRA; *E. coli* DNA—DNA content in µg per 1 mg CRA; LPS—endotoxin content in EU per 1 mg CRA. Mean ± standard deviation; each value based on four independent measurements (*n* = 4).

## Data Availability

The original contributions presented in this study are included in the article/Supplementary Materials; further inquiries can be directed to the corresponding authors.

## Supplementary Materials

The following supporting information can be downloaded at https://www.mdpi.com/article/10.3390/biom15121633/s1, Figure S1: pET-hmCRA plasmid map; Figure S2: Purification of CRA-cut by ion-exchange chromatography; Figure S3: Far-ultraviolet CD-spectrum of recombinant CRA and CRA-cut; Figure S4: Activity assays of recombinant CRA and CRA-cut; Figure S5: Quality assessment of the AlphaFold-predicted CRA model: pLDDT and PAE analysis; Table S1: Statistical significance of pairwise coagulation test (aPTT, PT, TT) comparisons between CRA protein variants and control.

## Abbreviations

The following abbreviations are used in this manuscript:

CRA	Cysteine-Rich Anticoagulant Protein
EU	Endotoxin Unit
CTM	C-terminal motif
CRA-cut	A Truncated Form of CRA Lacking the C-Terminal Motif
ATS	Antistasin
TT	Thrombin Time
PT	Prothrombin Time
aPTT	Activated Partially Thromboplastin Time
Hir	Hirudin
Chol	Cholesterol
PC	Phosphatidylcholine
PE	Phosphatidylethanolamine
PS	Phosphatidylserine
CD	Circular dichroism
CVD	Cardiovascular disease
IEX	Ion-exchange chromatography
