# Supplementary material for: Production, Purification, and Characterization of a Novel Cysteine-Rich Anticoagulant from the Medicinal Leech and the Functional Role of Its C-Terminal Motif
Source: Biomolecules. 2025 Nov 21;15(12):1633. doi: 10.3390/biom15121633 (PMC12731043; doi:10.3390/biom15121633)
Supplement: Supplementary file 1 [file biomolecules-15-01633-s001.zip › biomolecules-3976471-supplementary.pdf]

Supplementary materials

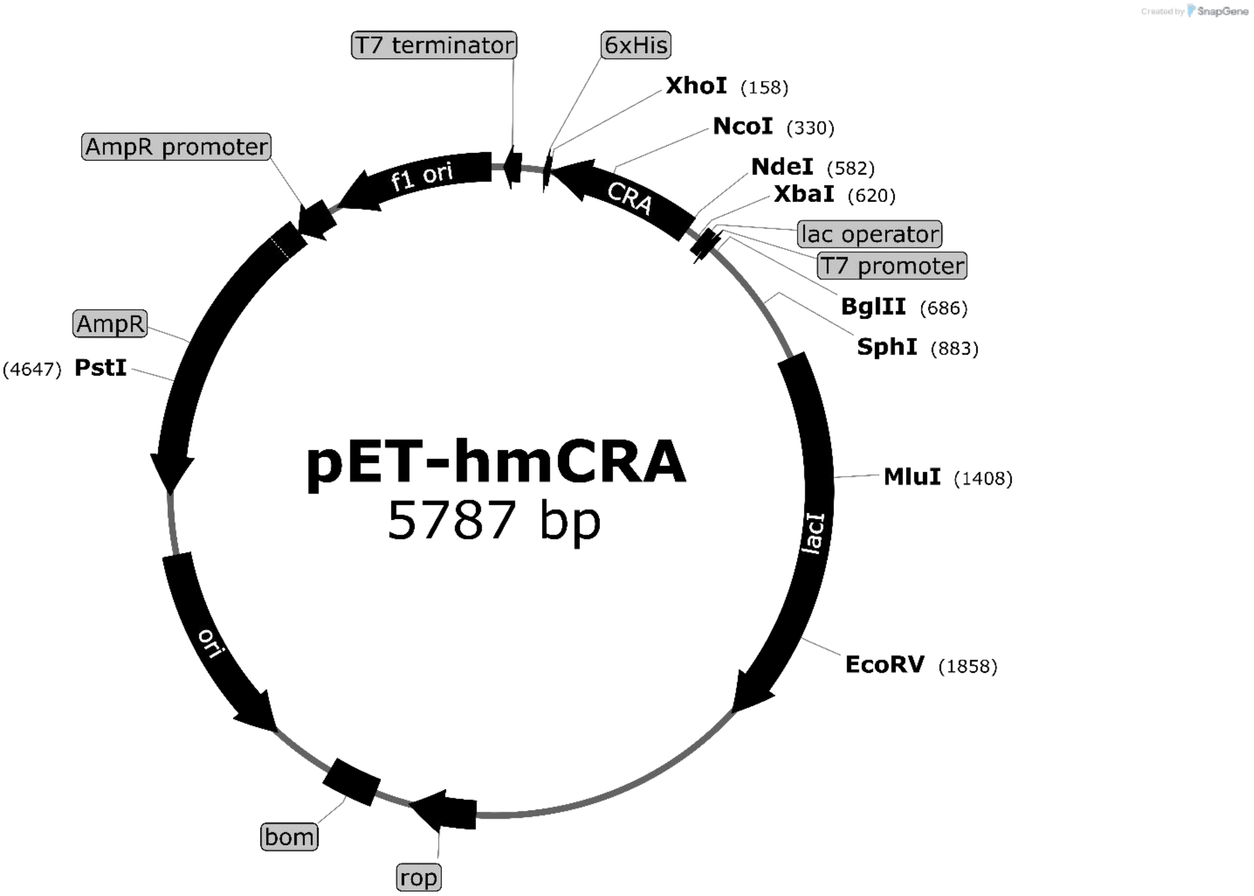

Figure S1. pET-hmCRA plasmid map. The plasmid carrying the gene encoding mature form of CRA fused to a C-terminal His-Tag was constructed using PIPE method [20]. The pelB signal peptide was omitted. PCR fragments were amplified from cDNA of *Hirudo medicinalis* salivary gland cells and assembled without ligation into *E. coli* TOP10 cells. Colonies carrying the target insert were selected and verified by Sanger sequencing. The truncated form, CRA-cut, lacking 28 C-terminal amino acids, was generated by PCR from pET-hmCRA, digested with XbaI and XhoI, ligated into the pET-22b(+) vector, and transformed into *E. coli*. The inserts were confirmed by sequencing.

20. Klock, H.E.; Lesley, S.A. The Polymerase Incomplete Primer Extension (PIPE) Method Applied to High-Throughput Cloning and Site-Directed Mutagenesis. *Methods Mol Biol* **2009**, *498*, 91–103, doi:10.1007/978-1-59745-196-3_6.

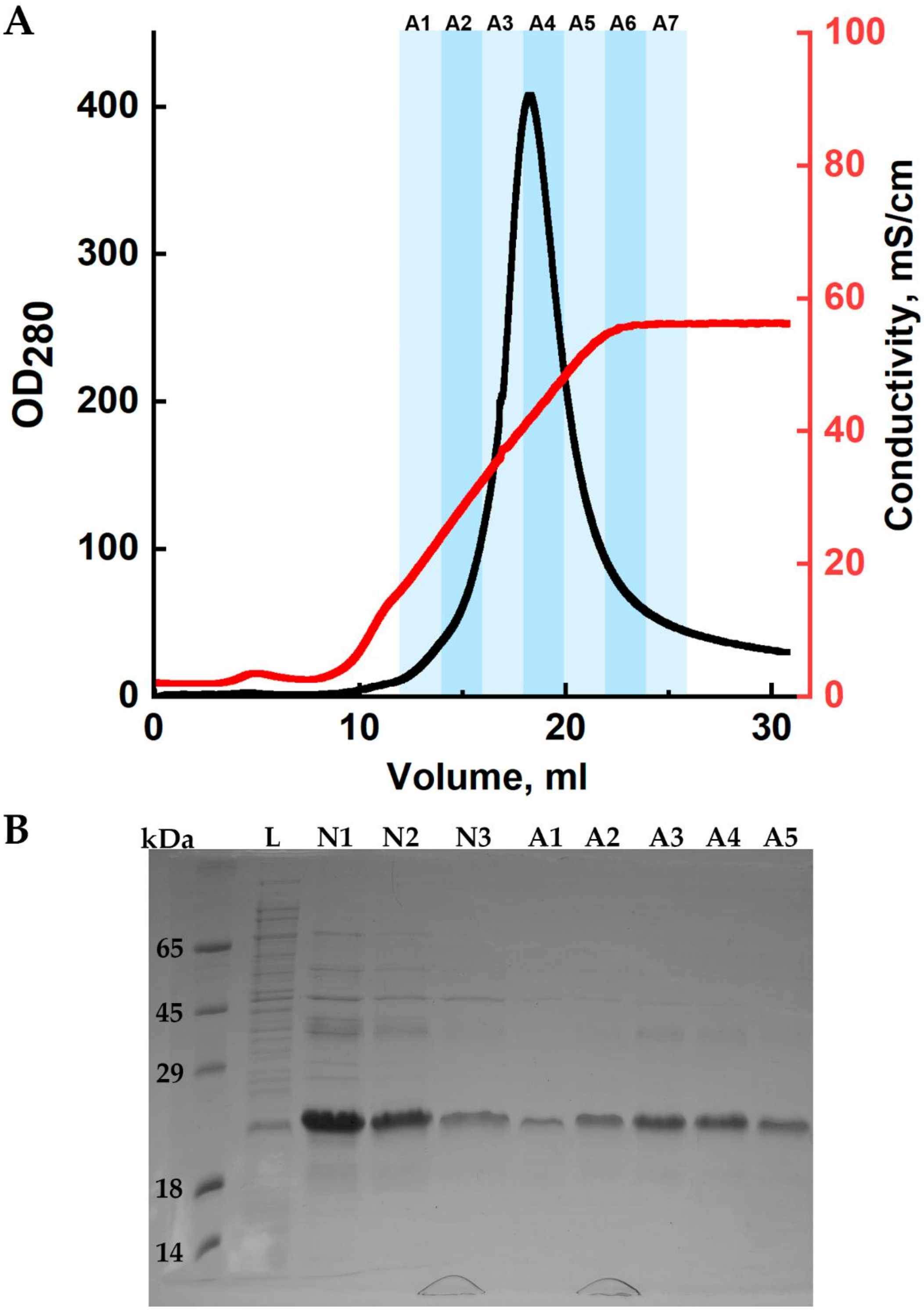

Figure S2. Purification of CRA-cut by ion-exchange chromatography. Cell lysate was clarified by centrifugation and heat treatment, and the supernatant was applied to a Ni-Sepharose column. After washing, the protein was eluted with imidazole-containing buffer, diluted, and further purified by ion-exchange chromatography. **A** – chromatographic profile: the x-axis represents elution volume, left y-axis represents optical density at 280 nm, and right y-axis represents conductivity. Fractions are indicated by shaded areas and labeled above (A1-A7, 2mL each). **B** – SDS-PAGE analysis of fractions with Coomassie G-250 staining. L – heated cell lysate; N1-N3 – metal chelate chromatography fractions, A1-A5 – DEAE chromatographic fractions, numbering corresponds to the chromatogram above.

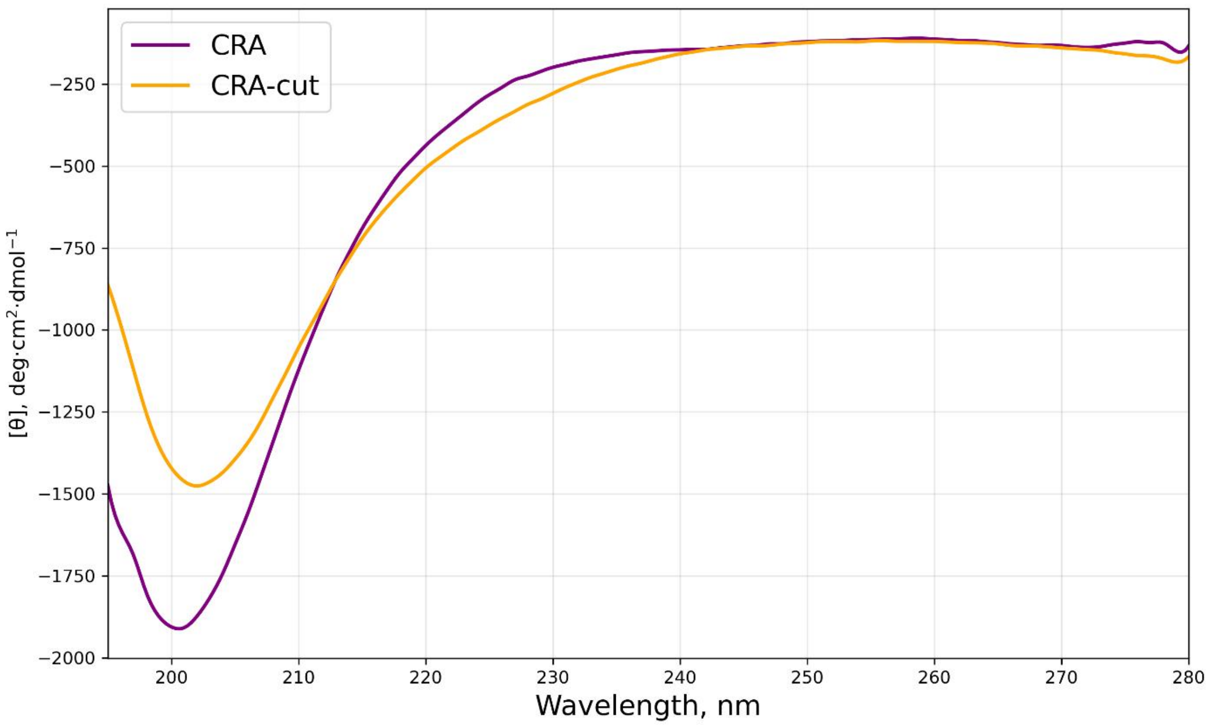

Figure S3. Far-ultraviolet CD-spectrum of recombinant CRA and CRA-cut. CRA and CRA-cut exhibit nearly identical CD spectra with a single negative peak at ~200 nm, indicating predominantly unordered structure with minimal α-helices or β-sheets. This is consistent with the loop-rich, disulfide-stabilized architecture of antistasin-family proteins. CD spectra were recorded at 0.2 mg/mL in H_2O using a Chirascan VX spectrophotometer (0.05 cm path length) and analyzed with CDNN software.

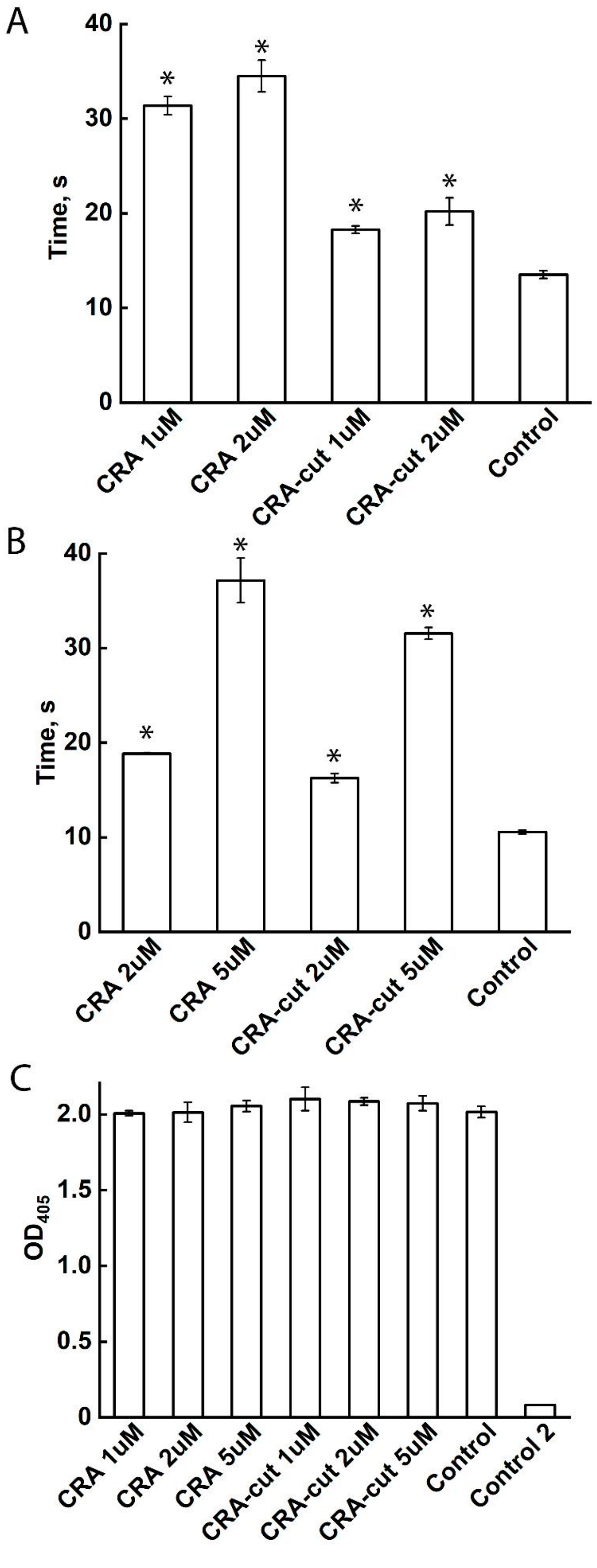

Figure S4. Activity assays of recombinant CRA and CRA-cut. **A** – the factor Xa clotting assay. For this, 100 µl of factor X-deficient plasma (X3) and 50 µl of 0.035M $CaCl_2$ was added to the cuvette and incubated at 37 °C for 1 min. Next, 25µl of sample and 50µl of factor Xa was added to the cuvette and incubated at 37 °C for 1 min. A clotting time score was derived for each sample. Data are presented as mean ± SD, n=4, samples, that are different from control sample at $p \leq 0.05$ are labeled with asterisk (*). **B** – the thrombin assay. For this, 25µl of recombinant protein and 75µl of fibrinogen (3.5 mg/ml) was added

into cuvette. After incubation for 2 min at 37 °C, 100µl of thrombin (3 IU/ml) reconstituted with standard imidazole buffer (Renam) was added. A clotting time score was derived for each sample. Data are presented as mean ± SD, n=4; samples, that are different from control sample at $p \leq 0.05$ are labeled with asterisk (*). **C** – the factor Xa chromogenic assay. For the this, 50µl of Factor Xa (9 mg/ml(17 nkat/ml)) (Renam, Moscow, Russia) and 50 µl of the test sample were added to the plate well and incubated at 37 °C for 5 min. Next, 50 µl of the 1.5 mM chromogenic substrate Acd-Leu-Gly-Arg-pNA was added and plate was incubated for another 5 min at 37 °C. The reaction was stopped with 100 µl of 50% acetic acid, and absorbance at 405 nm was measured using the AMR-100 microplate reader (Hangzhou Allsheng Instruments Co., Hangzhou, China). Control 1 – the assay was conducted without CRA or CRA-cut proteins. Control 2 – the assay was conducted without factor Xa.

Chromogenic activity assays for factors IIa, Xa, XIa, and XIIa.

For chromogenic assays, factors Xa, XIa, XIIa, or thrombin were incubated with a solution of CRA for 5 min at 37 °C, after which the corresponding substrate was added: S2765 for factor Xa; S2366 for factor XIa; S2302 for factor XIIa; and Tos-Gly-Pro-Arg-pNA for thrombin. The final concentrations of reagents in the reaction mixtures were as follows: Xa – 2.5 nM, S2765 – 200 µM; XIa – 2.5 nM, S2366 – 200 µM; XIIa – 2.5 nM, S2302 – 200 µM; thrombin – 2.5 nM, Tos-Gly-Pro-Arg-pNA – 200 µM. The anticoagulant activities of CRA were measured at two concentrations: 0.4 and 4 µM. The kinetics of p-nitroaniline formation were monitored using a microplate reader by measuring absorbance at 405 nm.

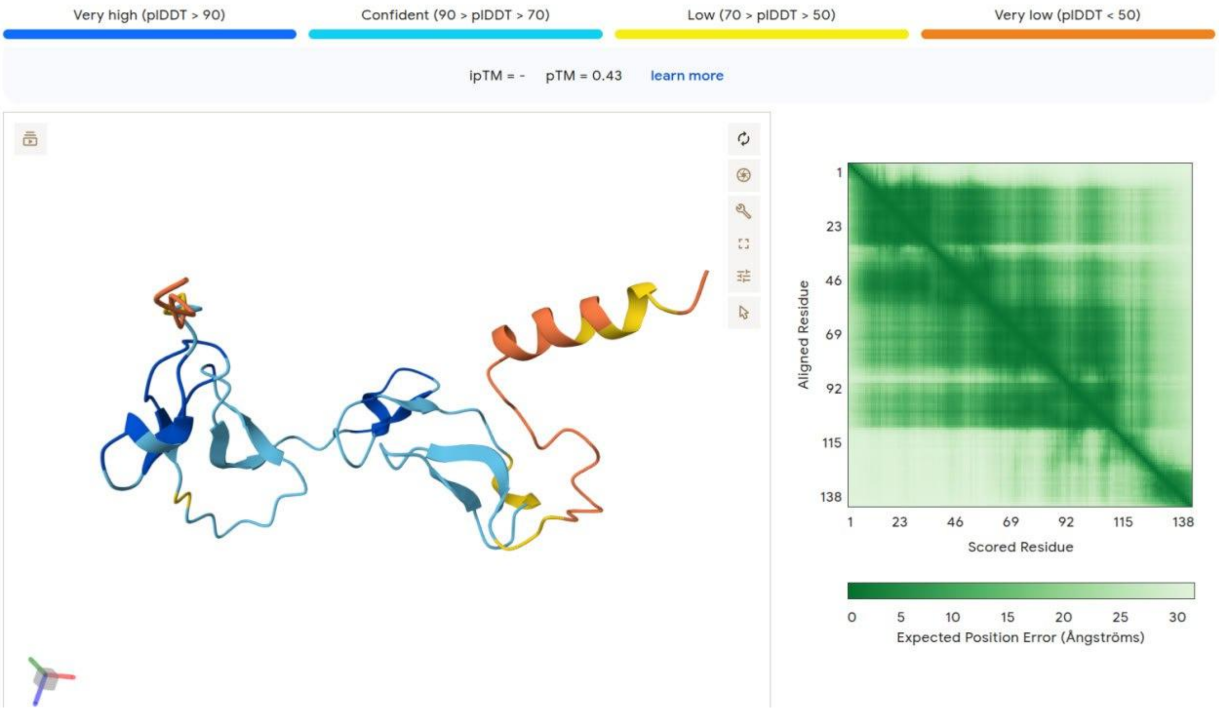

Figure S5. Quality assessment of the AlphaFold-predicted CRA model: pLDDT and PAE analysis. Each residue in the sequence is color-coded based on the model confidence score, pLDDT. PAE plot, showing regions of high confidence (dark green) and low confidence (pale green) for the predicted structure of CRA.

Table S1. Statistical significance of pairwise coagulation test (aPTT, PT, TT) comparisons between CRA protein variants and control.

APTT, 1μM	CRA P1	CRA P2	CRA P3	CRA-cut	C
CRA P1	1				
CRA P2	$5.3x10^{-3}$	1			
CRA P3	$2.2x10^{-2}$	$5.7x10^{-2}$	1		
CRA-cut	$3.6x10^{-3}$	$5.0x10^{-4}$	$2.4x10^{-2}$	1	
C	$3.4x10^{-3}$	$1.5x10^{-3}$	$1.8x10^{-2}$	$1.4x10^{-2}$	1

PT, 1μM	CRA P1	CRA P2	CRA P3	CRA-cut	C
CRA P1	1				
CRA P2	$2.0x10^{-6}$	1			
CRA P3	$2.7x10^{-5}$	$1.6x10^{-4}$	1		
CRA-cut	$1.0x10^{-4}$	$7.0x10^{-3}$	$8.6x10^{-4}$	1	
C	$4.0x10^{-5}$	$7.4x10^{-4}$	$3.9x10^{-4}$	$1.2x10^{-3}$	1

TT, 1μM	CRA P1	CRA P2	CRA P3	CRA-cut	C
CRA P1	1				
CRA P2	$2.4x10^{-2}$	1			
CRA P3	$3.5x10^{-1}$	$5.1x10^{-1}$	1		
CRA-cut	$5.0x10^{-2}$	$1.0x10^{-2}$	$8.7x10^{-3}$	1	
C	$2.8x10^{-2}$	$4.9x10^{-2}$	$3.6x10^{-2}$	$1.7x10^{-2}$	1

TT, 5μM	CRA P1	CRA P2	CRA P3	CRA-cut	C
CRA P1	1				
CRA P2	$2.8x10^{-2}$	1			
CRA P3	$9.2x10^{-2}$	$8.1x10^{-2}$	1		
CRA-cut	$1.1x10^{-2}$	$4.2x10^{-4}$	$2.3x10^{-4}$	1	
C	$1.4x10^{-2}$	$1.3x10^{-2}$	$1.1x10^{-2}$	$1.4x10^{-3}$	1

CRA P1, P2, and P3 – three independent CRA isolations , CRA-cut – a truncated form of CRA protein without C-terminal motif, C – control sample without protein. The p-values were calculated using Student’s t-test for independent samples, followed by Bonferroni correction for multiple comparisons. The table shows the adjusted significance levels. p-values < 0.05 after correction indicate statistically significant differences between the compared groups. Calculations were performed in Python 3.11.4 using the math and pandas libraries. Significant differences are highlighted in green, while non-significant ones are highlighted in yellow.
